# Personals

**Published:** 1918-04

**Authors:** 


### PERSONAL

**Announcement of removal, travel, and other matters of interest are requested. Please report errors in the listing of any physician in the State and other directories, that we may co-operate with the State Society in securing a correct list.**

---

Dr. Laurence G. Hanley spent two weeks in March in New York and Atlantic City.

Dr. Herman Johnson of Gowanda has returned from Fort Arthur, Baltimore, on a month's leave. He has been transferred from service in France to service in this country.

Dr. Frederick E. Strozzi of Buffalo has been commissioned First Lieutenant in the Medical Reserve Corps.

Dr. Benjamin Rogers has been appointed oculist for the Buffalo Division of the Wabash Railroad.

